# Evaluating collinearity effects on species distribution models: An approach based on virtual species simulation

**DOI:** 10.1371/journal.pone.0202403

**Published:** 2018-09-11

**Authors:** Paulo De Marco, Caroline Corrêa Nóbrega

**Affiliations:** 1 Dept. Ecologia, Univ. Federal de Goiás, Goiânia, GO, Brazil; 2 IPAM—Instituto de Pesquisa Ambiental da Amazônia, Belém, Pará, Brazil; Universita degli Studi di Napoli Federico II, ITALY

## Abstract

The increasing use of species distribution modeling (SDM) has raised new concerns regarding the inaccuracies, misunderstanding, and misuses of this important tool. One of those possible pitfalls − collinearity among environmental predictors − is assumed as an important source of model uncertainty, although it has not been subjected to a detailed evaluation in recent SDM studies. It is expected that collinearity will increase uncertainty in model parameters and decrease statistical power. Here we use a virtual species approach to compare models built using subsets of PCA-derived variables with models based on the original highly correlated climate variables. Moreover, we evaluated whether modelling algorithms and species data characteristics generate models with varying sensitivity to collinearity. As expected, collinearity among predictors decreases the efficiency and increases the uncertainty of species distribution models. Nevertheless, the intensity of the effect varied according to the algorithm properties: more complex procedures behaved better than simple envelope models. This may support the claim that complex models such as Maxent take advantage of existing collinearity in finding the best set of parameters. The interaction of the different factors with species characteristics (centroid and tolerance in environmental space) highlighted the importance of the so-called “idiosyncrasy in species responses” to model efficiency, but differences in prevalence may represent a better explanation. However, even models with low accuracy to predict suitability of individual cells may provide meaningful information on the estimation of range-size, a key species-trait for macroecological studies. We concluded that the use of PCA-derived variables is advised both to control the negative effects of collinearity and as a more objective solution for the problem of variable selection in studies dealing with large number of species with heterogeneous responses to environmental variables.

## Introduction

Species distribution modeling (SDM) is an interesting and efficient tool to deal with a variety of questions related to species geographic distributions [1–4]: What is the distribution of rare and/or endangered species? Which current areas should be turned into reserves to foster conservation of a species set? Where it is most profitable to search for new populations of a rare and little-known species? Which environmental factor controls and constrain the distributions of species or clades or ecosystems or biomes? What is the expected distribution of these species in the future, facing global climatic change? The list of questions is large, the reason why attention from scientific community continues increasing.

Nevertheless, as we accommodate this tool in our hands and our minds, we find several flaws, inaccuracies, misunderstanding, and misuses that affect its usefulness. Recent evaluations have revealed ineffective results due to incompleteness in occurrence datasets or environmental layers [5,6], uncertain due to modeling decisions [7–9], choice of climate variables [10], selection of background/pseudo-absence data [11–13], lack of transferability [14–16], inability or restrictive inclusion of biological interactions [17,18] or dispersal limitations [19,20], difficulties with prevalence estimations [21], spatial autocorrelation among occurrence points [22,23] or environmental predictors [24–26], ensembles of model results [8,9] and unstable model function resulted from collinearity in environmental datasets [27,28]. All these problems are important but some, such as uncertainty in model predictions, have received much more attention in the current literature. In this contribution, we add to the development of this tool by dealing with a less-analyzed factor, the collinearity of environmental predictors.

Every basic statistical text includes a big warning sign about effects of collinearity on regression model fit. Fitting regression models with collinear variables will generate large changes in estimated coefficients because partial regression coefficients are nonlinear functions of remaining collinear variables [29]. Thus, collinearity may produce unstable results since different random samples of the same datasets produce different estimated slopes. Consequently, collinearity is expected to increase uncertainty in model results. In a simulation study designed to analyze the importance of collinearity effects in ecological regression analysis, Graham [29] showed that even a low level of collinearity (on the order of r>|0.28|) may cause inaccuracies in model parameters and decreased statistical power. This effect is especially important since, when acknowledged by researchers using SDM, collinearity problems are frequently treated via analysis of pairwise correlation coefficients [30], followed by selection of only less collinear variables, using so different criteria such as R_spearman_<0.7 [30], R_pearson_< |0.5| [31], R_pearson_< |0.7| [32], R_pearson_< |0.8|.[33] or R_pearson_< |0.85| [34].

Numerous methods have been designed to deal with collinearity problems, but (surprising) they appear to be neglected by current statistical machinery applied to SDM and similar studies. Dormann et al. [27] reviewed those methods, showing considerable variation in performance among them; nevertheless, latent variable methods, such as use of the scores of a principal component analysis (PCA) in the regressions (usually known as principal component regression, PCR), presented good results. This set of methods is easily implemented and may overcome possible caveats that hinder use of collinearity-controlling methods in macroecological studies. Nevertheless, there is no detailed analysis of the collinearity effects on SDM or an evaluation of PCA derived variables for use in such studies. The only quantitative evaluation of uncertainty related to collinearity is the use of PCA and sequential regression explored by Dorman [35] in analysis of the distribution of Great Gray Shrike (*Lanius excubitor* L.) in relation to climate change. He found that model collinearity was of minor importance compared to other sources of uncertainty, but the analysis of only one species limits the generalization of these results. Otherwise, even if the original simulations of Dormann et al. [27] were not related directly to SDM techniques, they provide a comprehensive explanation of problems that emerge with collinear predictor variables, highlighting that no solutions exist to identify the correct causal relationships based on collinear predictors. However, particularly under latent-variable approaches, we rely only on a general expectation that a set of variables be “a proxy of some set of underlying ‘true’ relationships to unobserved or unavailable environmental variables” [27].

Considering the problem of choosing sets of environmental predictor variables for SDM, we must recall Austin’s [36] distinction between proximal, physiologically related, and distal, broad climatic variables in modeling. Even if proximal variables that are directly interpretable in terms of species niche requirements, collinearity among those variables and broad and easily-available climatic variables will help in producing effective model results [10]. Otherwise, it is still possible that strong collinearity may cloud the importance of a real proximal variable during any variable selection approach, affecting model results. Hence, collinearity among variables should be a key factor in SDM performance. Considering our incomplete knowledge on the real requirements of each individual species, this open the possibility that collinearity be a helper instead of an adversary to SDM improvement.

Dupin et al. [37] used principal component analysis to produce a new set of uncorrelated variables to model distributions of a widespread species. Hanspach et al. [38] used PCA-derived climatic variables to model distributions of European plant species. Serra et al. [39], Silva et al. [40] and Silva et al. [41] use the same procedure studying different aspects of bee species distribution in Brazil. More recently, Velazco et al. [42] evaluate of the use of climate and soil data for modelling neotropical plant species distribution also using PCA to control for collinearity. This procedure may be promising because, instead of dropping variables, which may contain useful or interpretable information about regional environment, it allows summarizing the broad axis of climatic variation across the study area, favoring both efficient prediction and interpretability. Since model performance and possible effects of collinearity on model performance may depend on a variety of incidental variables, with special focus on variation in environmental variables and individual characteristics of the species, a broader evaluation of PCA as an effective solution for this problem is required. We hypothesized that intrinsic model characteristics determine the sensitivity of SDM to collinearity effects, with the expectation that simpler (*i*.*e*. few parameters), presence-only methods will produce better results. Because collinearity increases model instability [27,29], we test whether use of PCA-derived variables, instead of the original set of collinear variables, can reduce variability in model performance measures. Nevertheless, intrinsic characteristics of the species directly affect its range size, prevalence, range geometry, and ecological/geographical marginality, all important features that affect SDM performance [43–46]. To deal with such complexity and to bring more generality to our evaluation, we use the “virtual species” approach [47] controlling key features of modeled species and environmental data.

In addition to pitfalls directly related to SDM development, other important sources of uncertainty are related to intrinsic species traits. Several of these issues are time- and space-dependent, and can drive or limit the species distribution in an (apparently) idiosyncratic way. These issues includes dispersal ability [19,20,48]; position in geographic space [45,49]; position in the ecological space (*i*.*e*. niche marginality), ecological tolerance or ecological specialization [43,50]; geometric properties such as range-cohesion, range-size, and prevalence [45,46,51–53]; and finally, non-equilibrium between species’ distribution and the environment [54]. All these issues can augment uncertainty in SDM predictions because they vary dramatically among species. Consequently, species’ distributions do not necessarily express their environmental requirements, and it is possible that species with identical niches may have very distinct distributions [55,56]. Otherwise, specific model settings may be more appropriate to model particular species in light of these differences [57], generating lack of standardization of accuracy between models. Even if we cannot control these issues, it is important to know that they exist and, eventually, to measure species’ idiosyncratic responses in SDM performance.

We distinguish here two different uses of SDM that directly affect the way those methods are interpreted or evaluated. First is the use to predict which of individual cells where expected the occurrence of a given species or to identify cells with high suitability to that species. This particular objective is shared by the majority of studies under a conservation biogeography approach such as reserve prioritization [58,59] with or without considering climate change issues [60]. The second is focusing on the range dynamics under a macroecological approach [54,61], which usually has a main focus on general patterns such as range-size variations among species [62,63]. Thus, good models for this approach are those that can predict the correct range-size independently than if individual cells are correctly predicted. As both approaches encompasses interesting ecological questions, our analysis was designed to deal with the accuracy of predictions for both the species distribution in each cell and of total range-size of modeled species.

## Methods

### Methods overview

Our general approach was to create realistic species distribution based on simulated ecological niches. The simulated distribution of each species is assumed as truth and, due to our simulation procedures, it represents both the environmental niche and the spatial restriction in colonization. Thus, it resembles better the general theoretical BAM scheme of species distribution [64,65] in which actual distribution is affected by the macro-climate variables that usually describes the Grinnelian niche of species [64] and the dispersal limitations determining the accessible are for species distribution [20]. Then, we produce spatial predictions of the distribution using common SDM procedures with original, collinear, climatic variables and non-collinear PCA-derived factors from the same climatic variable dataset. Finally, we compare the geographical prediction against the true spatial distribution of the species to evaluate the effects of model choices, model procedure and collinearity on SDM performance. Our approach based on geographical space allows to a proper evaluation if our SDM methods can estimate species distribution under those constrains.

### General simulation approach

We employed what has recently described as the “virtual ecologist approach” [47] to evaluate effects of collinearity among environmental variables on SDM results. This framework allows for the “evaluating of sampling schemes and methods, (statistical) analysis tools, model approaches and structures. Virtual data are generated by simulating (a) a virtual ecological model which includes key processes of the ecological system, (b) a virtual sampling model mimicking the observation procedure, (c) the methodological tools to analysis the ‘virtually’ observed data” [47]. Virtual species are seeing increasing use in SDM [66–69] mostly because they allow to separate the effects of individual features in complex models, which is never possible when using real species for comparisons. Moreover, evaluation of models for real species is subject to problems such as bias in sampling points [5,34], choice of environmental descriptors [70,71] and availability of appropriate independent sets of observations not affected by spatial autocorrelation in the environmental data [14]. Using virtual species allows comparing the resulting models with “true” distributions, overcoming these problems. Obviously, the success of this approach lies in the ability of the simulating procedure to mimic underlying processes in the range-distribution phenomena and the sampling points/SDM procedures.

**Fig 1:**
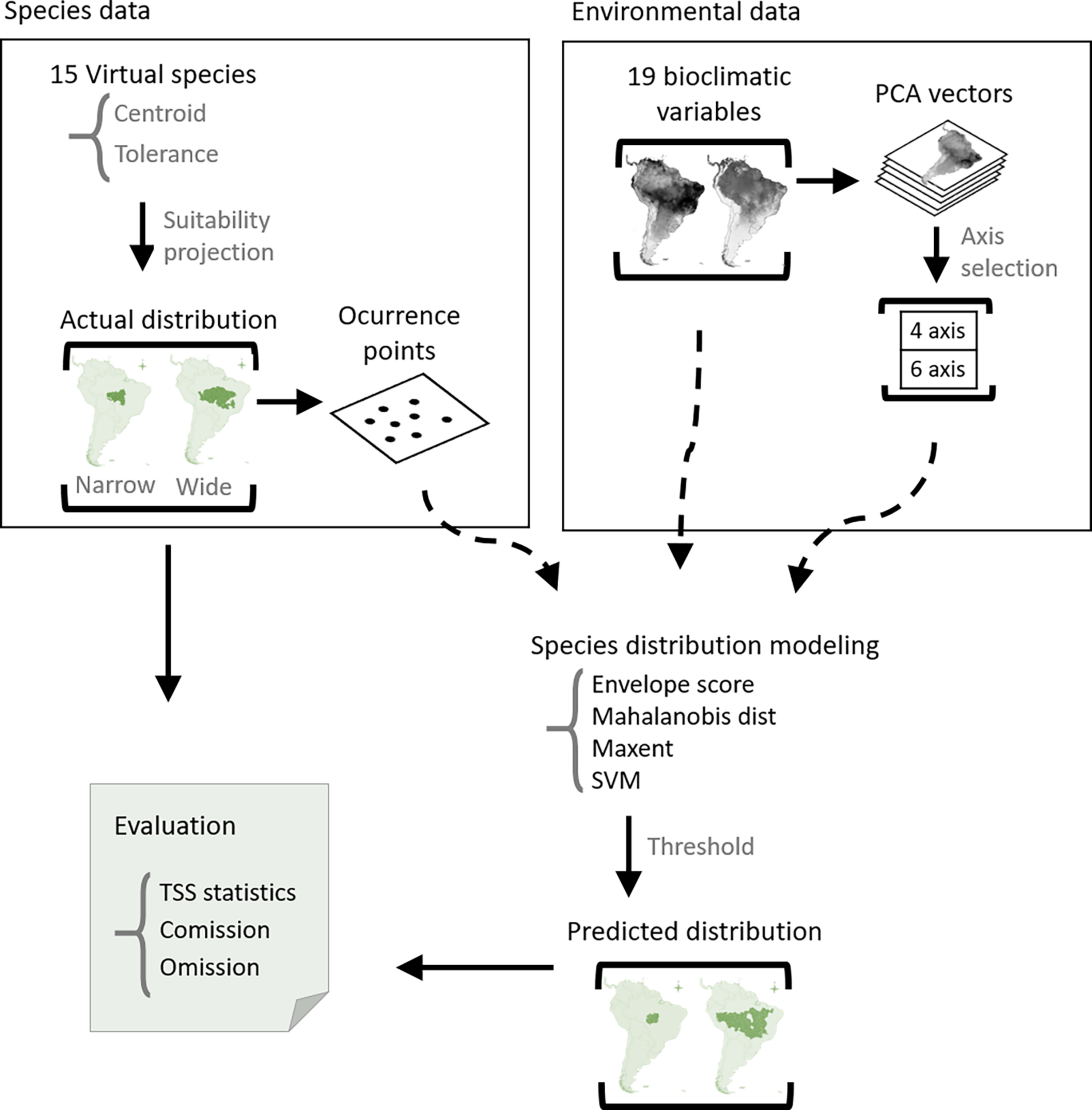
General simulation and analytical scheme to test the effect of collinearity among environmental variables on species distribution modeling results.

Our approach is based on (1) the definition of virtual species suitability in a given site as a function of the environmental variables and species Grinnellian niche, (2) its projection in the geographic space producing known distributional patterns, (3) sampling points in these distributions, (4) fitting SDM from these points using environmental datasets with and without controlling for collinearity and then, (5) comparing the results with the known distributional patterns. Fig 1 summarizes this process. One of the key aspects that contribute to the realism of these simulations is the process of projection of species distribution using a cellular automata model. Otherwise, each of these steps includes many parameters or methodological details that are described separately.

### Environmental data

The basic environmental data for our analysis were the 19 bioclimatic variables in WorldClim website (http://www.worldclim.org). Bioclimatic variables are derived from monthly temperature and rainfall values, and represent means and seasonal extremes of the environmental factors [72]. Many of these variables can be understood to play interpretable roles in constraining on the distributions of species and this dataset is used commonly in the literature [37]. We selected a spatial resolution of 10 arc-minutes (about 20 x 20 km cell size) across all South-America for analysis.

**Fig 2:**
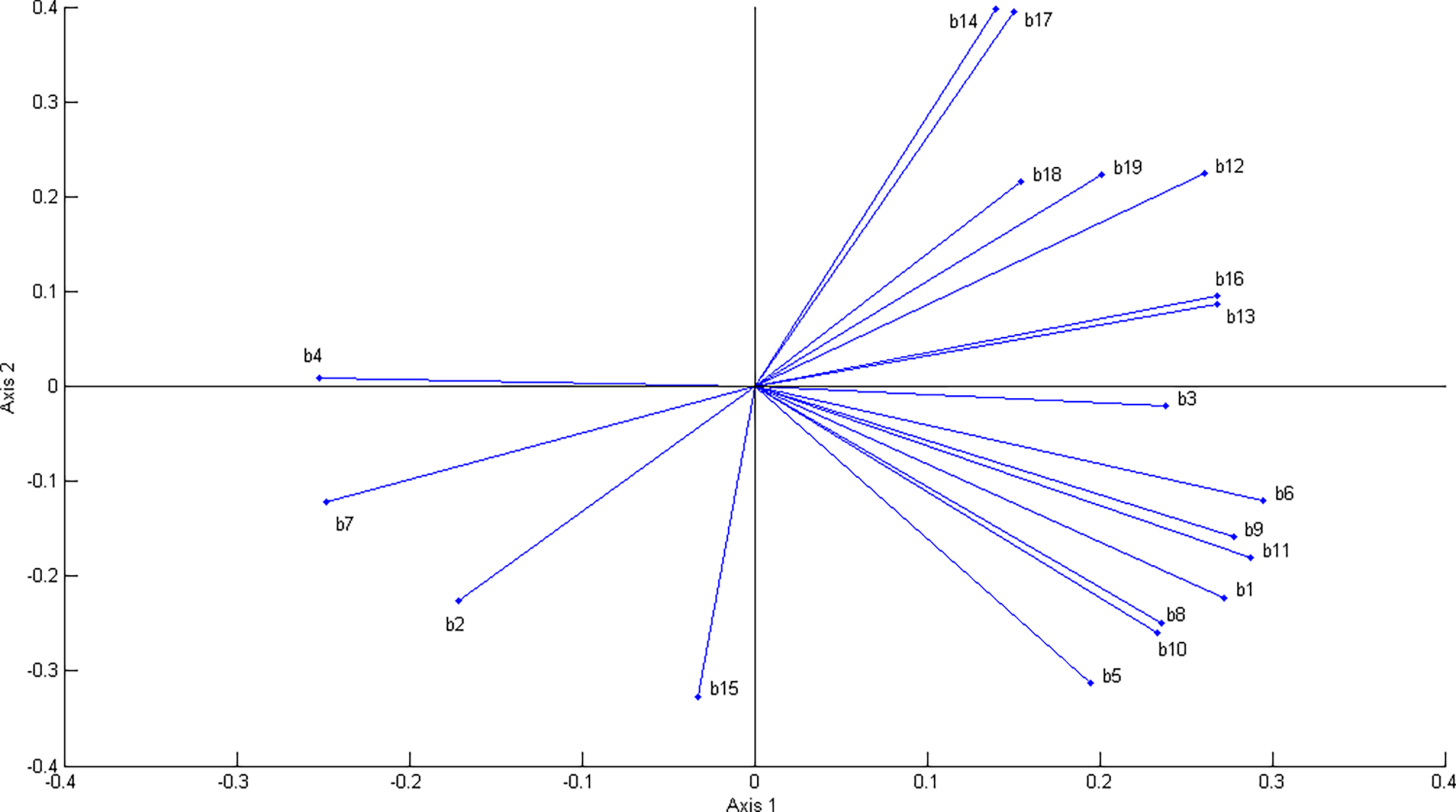
Factor loadings for the first two principal components of the Bioclim environmental variables for South America. Identifiers of the Bioclim variables: b1: annual mean temperature; b2: mean diurnal range; b3: isothermality; b4: temperature seasonality; b5: max temperature of warmest month; b6: min temperature of coldest month; b7: temperature annual range; b8: mean temperature of wettest quarter; b9: mean temperature of driest quarter; b10: mean temperature of warmest quarter; b11: mean temperature of coldest quarter; b12: annual precipitation; b13: precipitation of wettest month; b14: precipitation of driest month; b15: precipitation seasonality; b16: precipitation of wettest quarter; b17: precipitation of driest quarter; b18: precipitation of warmest quarter; b19: precipitation of coldest quarter.

We calculate a principal component analysis (PCA) based on the correlation matrix of the environmental variables to derive new uncorrelated variables for the study, using standard procedures [73]. This analysis illustrates the high level of collinearity among the original climatic layers since the first four axes retain 90% of the variation in the overall dataset (Table 1). Examination of the first two PCA loadings reveals some of the strong correlations among variables in the dataset (Fig 2), most of the variables were positively related to the first axis, but temperature seasonality (b4) and temperature annual range (b7) were negatively associated. The most important variables for the second axis were related to the precipitation with positive loadings for precipitation in the driest month (b14) or quarter (b17) and negative loadings for precipitation seasonality (b15). Indeed, some variables had almost identical positions on the first two axes, suggesting high collinearity in pairwise comparisons (*e*.*g*. mean temperature in the wettest quarter (b8) and warmest quarter (b10); precipitation of the wettest month (b13) and wettest quarter (b16); precipitation of the driest month (b14) and driest quarter (b17).

**Table 1:** Eigenvalues and proportion of total variance explained by each axis derived from a principal components analysis of climate data for South America.

Axis	Eigenvalue	Proportion (%)	Cumulative proportion
PC1	10.42	54.84	54.84
PC2	3.86	20.30	75.14
PC3	1.71	9.02	84.16
PC4	1.14	6.01	90.17
PC5	0.74	3.92	94.08
PC6	0.49	2.56	96.64
PC7	0.29	1.51	98.16
PC8	0.15	0.77	98.92
PC9	0.11	0.56	99.49
PC10	0.05	0.27	99.76
PC11	0.02	0.08	99.84
PC12	0.01	0.07	99.91
PC13	0.01	0.03	99.94
PC14	0.00	0.02	99.97
PC15	0.00	0.02	99.98
PC16	0.00	0.01	100.00
PC17	0.00	0.00	100.00
PC18	0.00	0.00	100.00
PC19	0.00	0.00	100.00

Many criteria could be used to select a representative set among PCA-derived variables [74], with the choice among them affected by sample size, correlation structure, and departure from normality [75]. Since this choice may affect the SDM results, we also evaluated the use of different criteria for model performance. We chose to use two simple criteria that are conservative, extrapolating the number of dimensions needed to describe the correlation matrix [74,75]. Regardless, this step is consistent with our objective in modeling species’ distributions, trying to include as much as possible environmental information. One criterion was to retain the axis with latent root higher than one (*i*.*e*. the Kaiser-Guttman criteria), which selected the first four axes in our analysis (Table 1). The second was to retain the set of components that explained at least 95% of the total variance (*i*.*e*. fixed cumulative eigenvalue criteria), which selected the first six axes (Table 1). PCA scores of each cell were calculated using the eigenvectors; Layers were re-projected into the geographic space as *ASCII* files for further analyses (this dataset is available from the corresponding author).

### Species distribution simulation

We follow the general approach of De Marco et al. [54] to generate geographic distributions of the virtual species. This approach includes: (1) definition of a centroid in ecological space, (2) establishing tolerance values, (3) setting the relation between suitability and environmental values in ecological space, (4) projecting suitability into geographic space, and (5) simulating species’ distributions based on suitability using a spatially explicit automata cellular model. This protocol was designed to be consistent with current view of distribution of species as a function of intrinsic traits that limit position and tolerance in niche space, but also regarding spatially explicit dispersal constraints [20,65]. Hence, we echoed Austin [36] in emphasizing that generating artificial data or virtual species’ distributions must reflect an existing theory about the whole process.

The ecological space for this study comprised the same climatic variables described above. We modeled suitability for each virtual species as a Gaussian multivariate distribution function of the basic environmental variables [67,69,76]. This approach allows evaluating possible influences on final SDM accuracy due to differences in relative positions of the optimal environmental combination (*i*.*e*., the centroid in environmental space) and the environmental tolerance (the variance of the Gaussian function). Fifteen centroids were chosen to represent a variety of environmental conditions in South America, and the different biomes present there. For each centroid, we chose two tolerance values representing narrow and broad tolerances. We used previous simulations of variance values to guide choice of actual tolerance values. We finally selected 20% of the variance of each environmental variable in South America for the narrow tolerance species and 60% for the broad tolerance species (description of the centroids of each species in environmental space is provided in S1 and S2 Tables).

The Gaussian curve was re-scaled to have a value of 1.0 for the optimal environment (centroid). This assures that species will be persistent on optimal environments, reducing stochasticity in optimal environments and, thus, reducing internal fragmentation in range during simulations. This relation was then projected as a deterministic suitability map in the geographic extent of the environmental variables, and treated as the input for the cellular automata model. The suitability of a given cell did not change during the simulation. A seed point, representing the center of origin of the virtual species’ distribution, was selected among the points with highest suitability. Whether attributable to this cell a value of one, meaning that the species is present there. The automata model was based on a simple colonization-extinction model evaluated iteratively for each cell. First, for each occupied cell, environmental suitability was converted directly into a probability of persistence to the next time step. To reduce variability in both low and high suitability sites, occupancy in cells with <0.1 suitability were always set to zero and those >0.9 were always set to one. These thresholds reduce the possibility of of instable distribution in high-quality habitats or colonization of poor-habitats. In previous tests of this simulations, the absence of such thresholds produced less stable distributions and high fragmentation of species-ranges. Otherwise, populations in a cell of suitability of *p* had a probability *1-p* to gone extinct. The second component was colonization: an occupied cell provides invasion propagules that can colonize nearby cells (1 cell distant) or ‘jump’ to cells 2–5 cells apart. As such, the species can pass through unsuitable areas up to 4 cells wide. The number of cells traveled by each propagule followed a uniform distribution with three dispersal attempts allowed by iteration. The dispersal process here was different from De Marco et al. [54], but was more realistic since it allowed long-distance dispersal. If the target cell is unoccupied, it may be colonized based on the same random test related to its suitability described for the extinction process. Rescue effects are allowed since colonization is evaluated before the extinction process.

**Fig 3:**
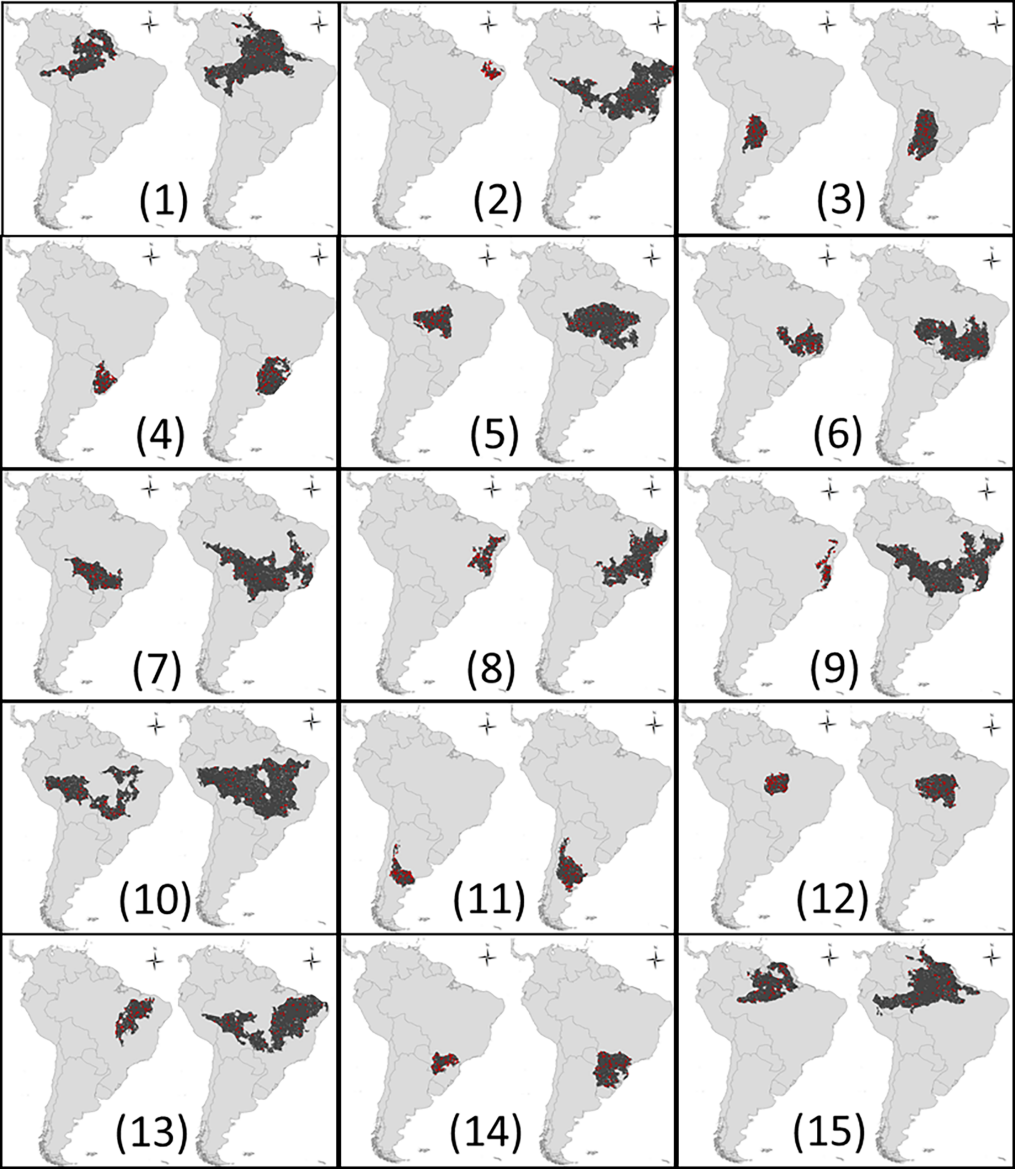
Virtual species used in simulations. In each cell, the two maps represent species with the same niche centroid but with small and large tolerance in ecological space, respectively. The numbers will be used to identify each species for the rest of this paper.

The resulting distribution for each species is thus a function of the intrinsic properties of the species, which are controlled by our experimental design, but also by availability and distribution of suitable areas in geographic space. This caused some broad-tolerance species to differ only subtly from its corresponding narrow-tolerance species (see species 4, in Fig 3). On the other hand, the difference in the range size observed in geographic space may be greater than that predicted based only on its tolerance (see species 2 in Fig 3). Both cases may represent the realism of our simulations and highlights the importance of distinguish broad/narrow tolerance in environmental space from broad/narrow ranges in the geographic space for interpretation of the resulted models.

### Sampling occurrence points

The number of sampling points relates directly to the representativeness of the climatic niche in a species for the modeling process. Effects on efficiency of modeling algorithms are well-known, with documented tendencies toward to overfitting in Maxent [28,77]. For each species, we selected a set of 50 points at random among the occupied cells at the end of the range-distribution simulation. We repeated the process 10 times allowing evaluation the uncertain related to different samples from the same species range, which is essential to the analysis of model instability related to collinearity.

### Species distribution modeling techniques

We used four different modeling techniques to generate species distribution models: Envelope Score (ES), Mahalanobis Distance, Maximum Entropy (Maxent) and Support Vector Machines (SVM). They are selected to represent a spectrum of model complexity [52], and different ways of using information from the occurrence points. Envelope Score and Mahalanobis Distance are true presence-only methods while Maxent and SVM uses background information during the model fitting process [21]. We expect that those differences may influence the collinearity effects on SDM performance.

Envelope Score is the simplest algorithm used in this study, since it is a quantitative version of the Bioclim algorithm, which describes rectilinear bioclimatic envelopes around occurrence points in the environmental space. Envelope Score was implemented using OpenModeller Desktop version 1.1.0 [78]. Mahalanobis Distance, the second simplest algorithm, is an environmental distance method that controls the covariance between variables at occurrence points. As a result, it requires more occurrence points than environmental variables; since the matrix inversion cannot be done using standard methods when the reciprocal condition of the covariance matrix is lower than 1. In such cases, we estimate the Mahalanobis distance using the pseudo-inverse of the covariance matrix.

Maxent version 3.3.3e [79,80], is considered one of the most efficient algorithms [81,82] and certainly among of the most used. Maxent can be adjusted based on several parameters, including choice of the feature types, number of background points, and the regularization parameter. The features types control the possible relationships between the occurrence data and the environmental variables, allowing for linear, quadratic, product, hinge and discrete responses. More features demand increased number of parameters and elevate the possibility of overfitting. In this paper, we follow Anderson & Gonzalez [57] by comparing the Maxent default (MXDEF) to a tuned model settings aiming to reduce model complexity by allowing only linear+quadratic (MXLQ) features. Due to internal settings of Maxent, this choice cause the use linear+quadratic+hinge features for our models.

The last algorithm used was SVM (Support Vector Machines), which is characterized as a group of supervised learning methods. This algorithm maps input vectors into a higher dimensional space by finding a hyperplane that separates presence and pseudo-absence records. We used the standard algorithm (C-SVC regularized support vector classification) with a radial basis kernel function as implemented by the OpenModeller.

Both Maxent and SVM models depends on the choice of background points. In all cases we choose 10000 random points in the geographical extent of South America as background for model fit.

Models for each virtual species were developed with each SDM algorithm using all raw environmental variables (RAW) and the two sets of uncorrelated PCA-derived variables: four PCA axes (PCA4) and six PCA axes (PCA6).

### Evaluating binary predictions

Our evaluation procedure was based on comparison of real distribution of the virtual species and a binary prediction of the models. We apply a threshold that maximizes the sum of sensitivity and specificity to minimize both omission and commission errors, and could be derived from the ROC curve. This will be referred hereafter as balance threshold. The balance threshold is the most commonly used method in practical applications [83,84], therefore it was used here to the core evaluation of the collinearity effects. Otherwise, only for the comparison of estimated range-sizes we also apply the “least training presence threshold” (LPT), which assumes that species must occur in locations equally or more suitable than those at which it has been found. Thus, LPT threshold is designed to only minimize omission errors. As the LPT is expected to generate larger distributions, we compared its accuracy to estimate the overall range-size with the balance threshold. Although there are other ways to derive range-size, we choose simply to use the estimate derived from the thresholding the suitability map. These estimates are simpler and frequently used in practical studies of conservation biology [85–87]. Moreover, they represent known methodological constrains related to omission and commission errors that will be useful to evaluate the factors affecting model performance.

To evaluate the models we used the overprediction rate (OP) [88], underprediction rate (UP) [= false negative rate 89], and the composite index True Skill statistics (TSS) that performed more effectively than other evaluation measures [90]. We choose to use the basic measures of model performance related to the omission (UP) and commission (OP) together with a synthetic measure (TSS) following Lobo et al [91] about possibly misleading results of synthetic measures. TSS was hypothesized to be less sensitive to prevalence, a common problem in such measures [92], including the more commonly used AUC [93]. Nevertheless, there is a lot of evidence that TSS is always affect by prevalence (our simulation results; Leroy, B et al., pre-print https://www.biorxiv.org/content/early/2018/05/11/235770). Thus, we try to include prevalence as a covariate in our analytical procedures. This choice suited our need to control for prevalence since we are comparing the results to its “true” distribution but may not function for analysis SDM exercises with real data. Another general problem with evaluation measures is the requirement of pseudo-absences data; we did not have that problem thanks to our virtual species, with complete knowledge of their presence and absence. Hence, model evaluation was achieved by comparing the actual and predicted distribution after thresholding.

### Statistical analysis

The main hypothesis in this study is that using PCA axes may produce better models, than those based on original variables. This increased accuracy may be affected by model procedures, species’ tolerance in environmental space (“narrow” and “wide”), and individual species real prevalence. Prevalence is calculated as the ratio of number of occupied cells of each virtual species simulations and the number of cells of geographical extent in the analysis. Inclusion of prevalence was especially important to control of its effects on our response variables. We may also expect interactions among those explanatory variables. For instance, some procedures may be more effective for restricted than for broadly distributed species [94–96]; hence, most relevant issues in this analysis refer to the existence of interactions among those variables [97]. We used a three-factor (type of environmental variables, algorithm and tolerance) ANCOVA with each species prevalence as a covariate. We explored significant interaction terms of the ANCOVA with a special caution considering the large degrees of freedom that resulted from simulation procedures, giving our tests high statistical power. Thus, we used confidence interval estimates to support the more important conclusions from these analyses.

We also evaluated effects of collinearity on model stability by estimating the variance of the TSS as a quantitative measure of the variability among sub-samples of occurrence points. We estimated this quantity for each virtual species, sampling effort and SDM algorithm and compared among the raw variables and 4 PCA and 6 PCA. For these analysis, we used inferences based on 95% confidence interval estimates.

Finally, as observed earlier, variation of the environmental variables in geographic space may affect the conversion of environmental tolerances into geographic ranges. Hence, tolerance alone may not be a good surrogate for range size, while range size alone may be an important factor affecting model accuracy. Thus, we tested accuracy of the models by a standard least-squares linear regression between predicted and real species range size. “Good” models should produce a close relationship. Models with high R^2^ with intercept near zero and slope near 1 are good predictors of absolute range size values. Nevertheless, if R^2^ are high but the other criteria are not met, the model could be considered still a good predictor of relative differences among range sizes.

## Results

The interaction of all of our main explanatory variables were statistically significant for TSS, but not for OP and UP (Table 2). Nevertheless, all other interactions were significant. This result highlights the complexity of species responses as regards predictive ability and on the choice of the best algorithm for modelling. The importance of prevalence was clearly observed affecting OP, UP and TSS, but its explanation power vary widely. Prevalence explains only 2,6% of the variation of OP but explains 36% of variation in UP and 56% of variation in the TSS in our experiment. This support our initial use of this covariate, but create a special concern about studies that do not control for this variable in the evaluation of the predictive ability of SDM procedures.

**Table 2:** Results of the factorial ANCOVA for the Overprediction (OP), Underprediction (UP) rates and TSS measure as dependent variables calculated under the balance threshold. DF is degrees of freedom.

		OP		UP		TSS	
	DF	F	p	F	p	F	p
Prevalence	1	30	<0.001	1398	<0.001	2843	<0.001
Environmental Vars (ENV)	2	453	<0.001	1272	<0.001	970	<0.001
Algoritm (ALG)	4	58	<0.001	790	<0.001	805	<0.001
Tolerance (TOL)	1	505	0.427	0.6	0.427	24	<0.001
ENV*ALG	8	23	<0.001	308	<0.001	294	<0.001
ENV*TOL	2	40	<0.001	9	<0.001	5	0.010
ALG*TOL	4	22	<0.001	51	<0.001	45	<0.001
ENV*ALG*TOL	8	1	0.247	1	0.219	3	0.016
Error	4469						

**Fig 4:**
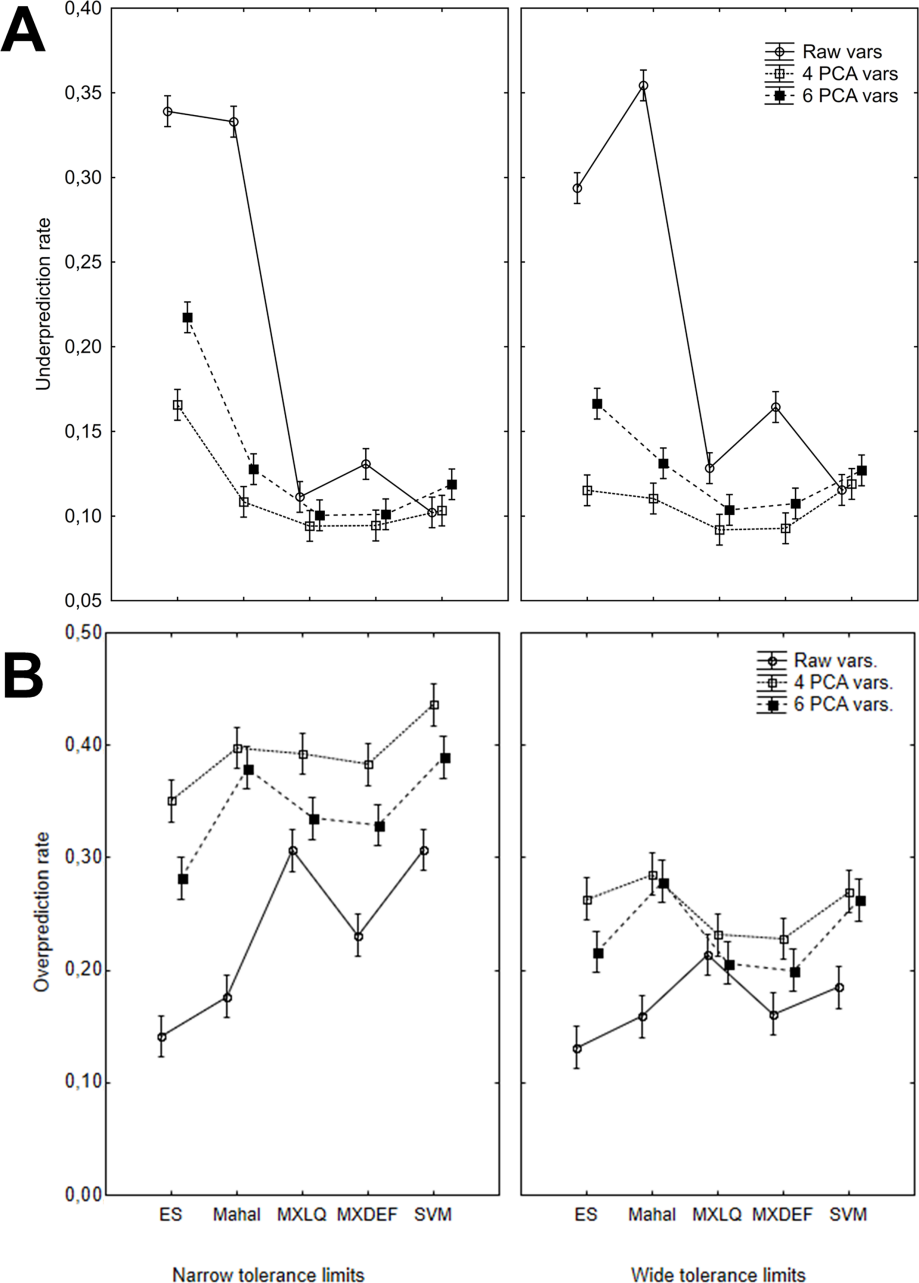
Interaction plots for the effect of environmental variables, modeling algorithm, and species tolerance in the environmental space on the Overprediction (A) and Underprediction (B) rate of the distribution models using balance threshold. Bars represent confidence intervals of 95%.

Considering the observed interaction in the analysis of all our response variables, we present all 3-interaction plots to allow a better understanding of the observed patterns. Considering only the results of the Underprediction rate with raw collinear variables (Fig 4A) it is easy to discriminate ES and Mahalanobis (hereafter low accuracy models) with UP higher than 0,3 from MXDEF, MXLQ and SVM (high accuracy models) with UP values never higher than 0,15. The use of PCA-derived variables reduce the UP for all algorithms, except for SVM which appears relatively unaffected by the collinearity in environmental variables. In general, the lowest UP values are observed for the use of 4-PCA variables. Moreover, the general patterns appear similar from narrow and wide tolerance species simulations. An almost opposite pattern is observed for the Overprediction rate (Fig 4B). First of all, the highest OP are observed for 4-PCA followed by 6-PCA variables, with the raw collinear environmental dataset as the most accurate model under this measure. There are less difference among algorithms, but ES and Mahal presented the best results. Otherwise, there a clear higher OP for species with narrow tolerance compared to wide tolerance.

**Fig 5:**
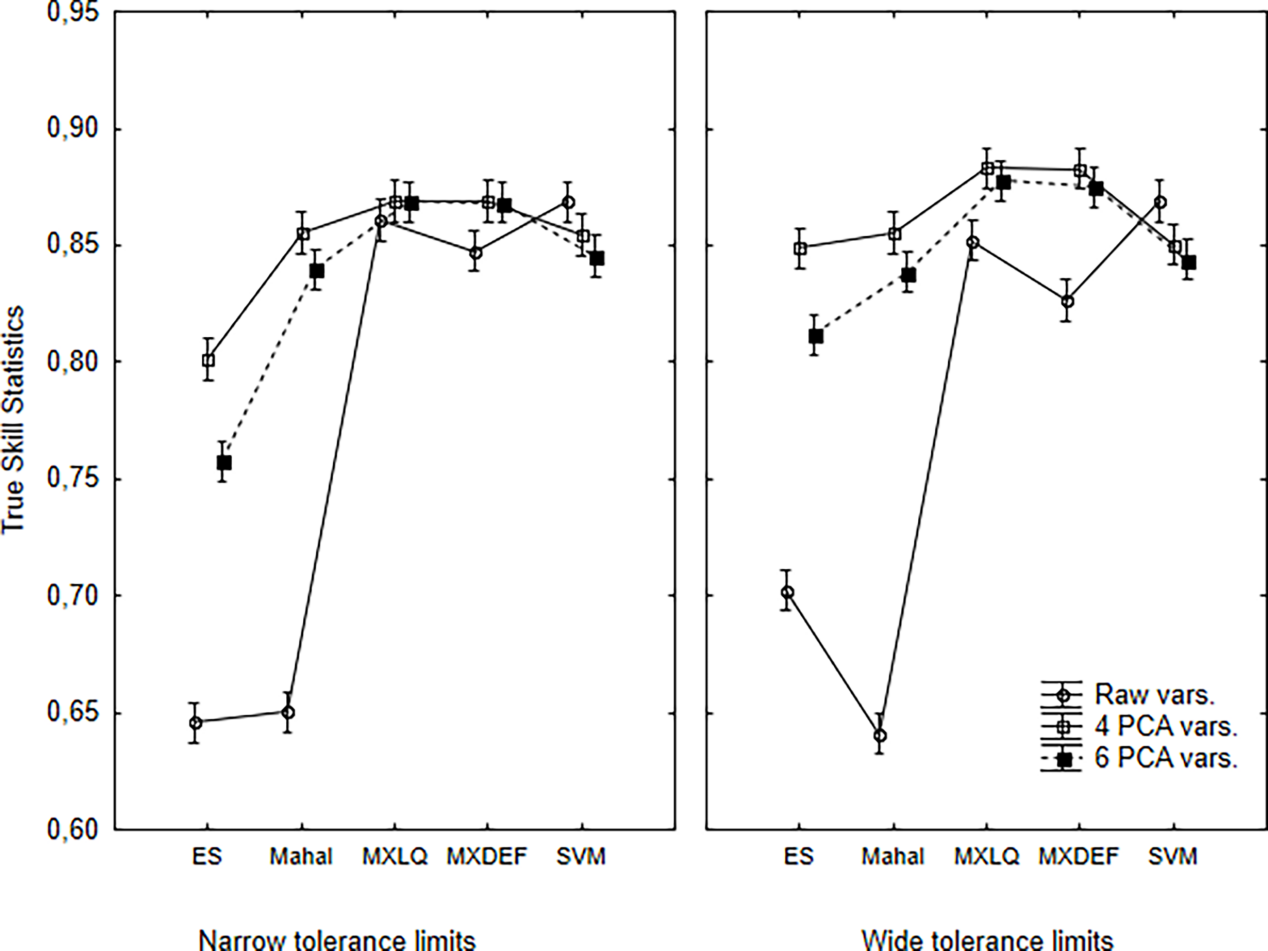
Interaction plots for the effect of environmental variables, modeling algorithm and species tolerance in the environmental space on the TSS of the distribution models using balance threshold. Bars represent confidence intervals of 95%.

Taking in account the interpretation above of the OP and UP, the composite measure TSS, may provide some confuse signs (Fig 5). TSS shows the distinction between low (ES and Mahal) and high (Maxent, SVM) accuracy models, most drived by the UP results. Clearly, the use of non-collinear variables produced best models, but this improvement is most observed for low accuracy models. SVM appear unaffected by collinearity or may perform slightly better with raw data. For Maxent models, the increase in TSS values is higher for wide tolerance species.

**Fig 6:**
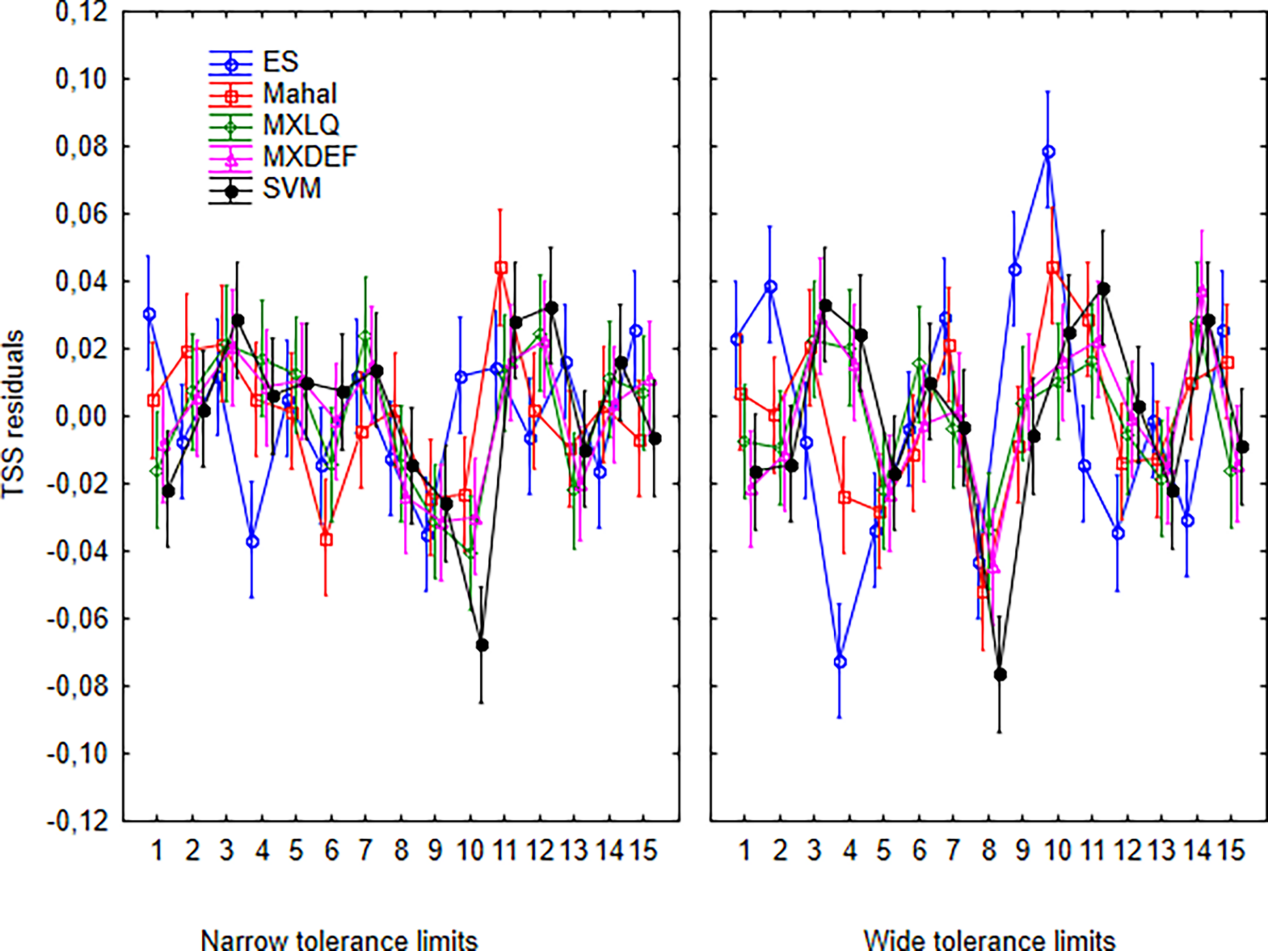
Residual plot of the ANCOVA results of the TSS response variable in relation to modelled species, modeling algorithm, and tolerance in environmental space. Bars represent confidence intervals of 95%.

Idiosyncrasy–a variable response of individual species to model procedures—appears slightly higher for wide-tolerance than for narrow-tolerance species (Fig 6). It is possible to observe higher variation of the residuals in relation to different algorithms for the same species in Fig 6, which means that no single algorithms has a superior performance over all modelled species.

**Fig 7:**
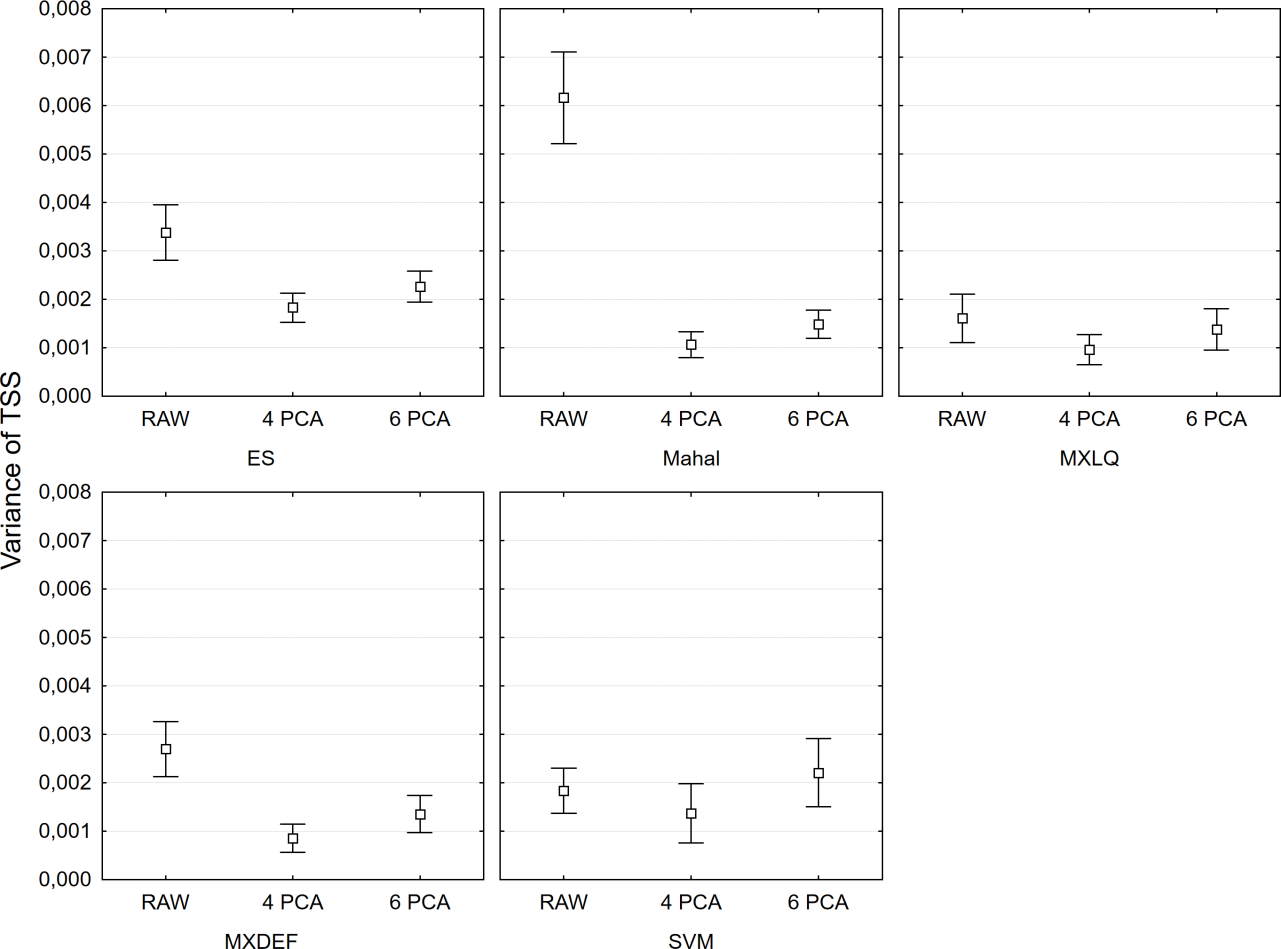
Variance of the TSS measure for different SDM algorithms with raw environmental variables and the first four PCA axis (4 PCA) and six PCA axis (6 PCA). Bars represent the 95 confidence intervals.

**Fig 8:**
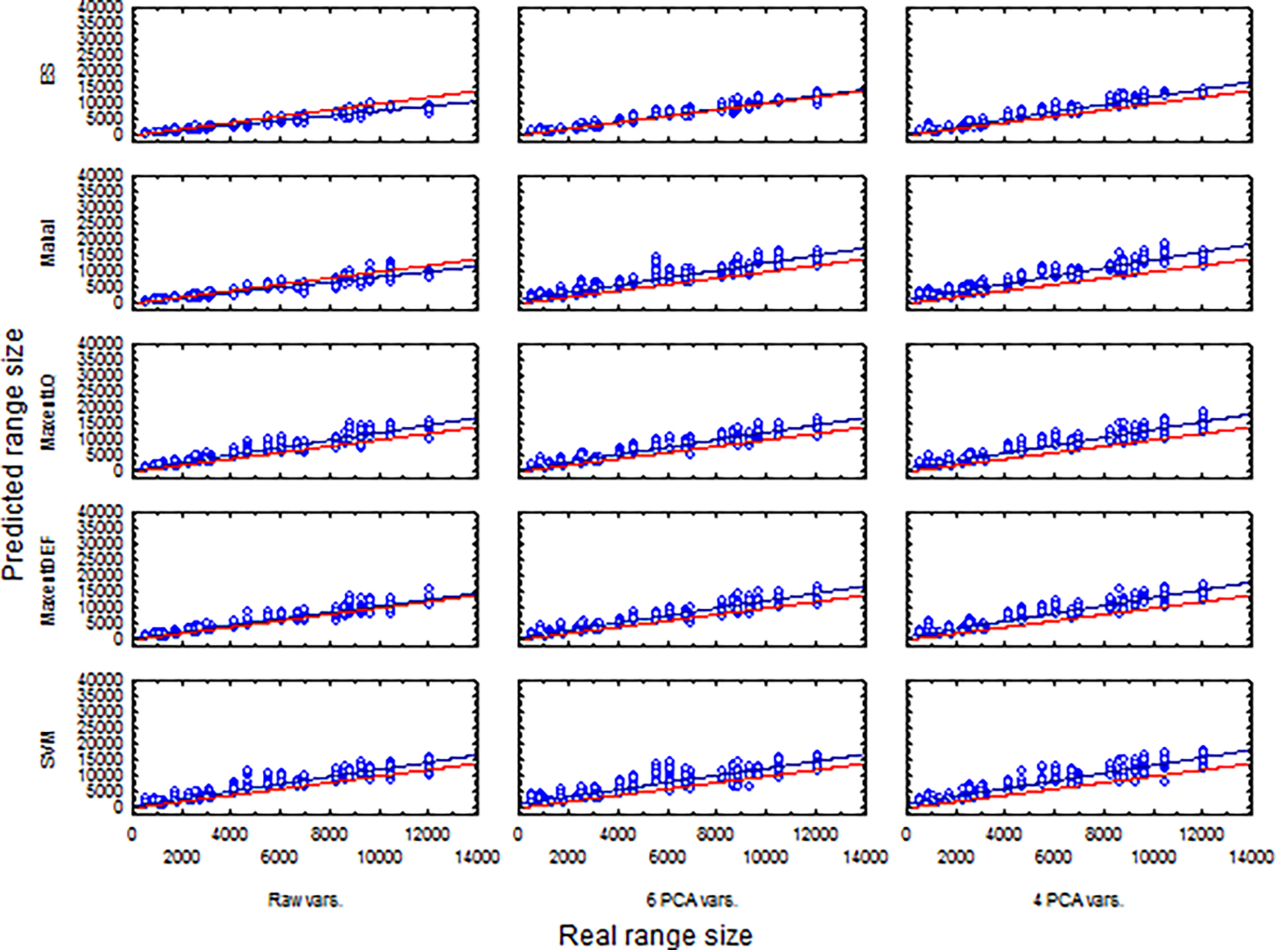
Linear regression of predicted on real range size for modeled species using Balance thresholds. Red lines represent the linear regression of the data; blue line show the prediction of the relationship if the predicted range-size were equal to the real range-size.

The results of models support the claim that a collinear set of variables increase model instability, especially for ES and Mahalanobis, but also for MXDEF (Fig 7). Range-size linear regression predictions yielded good agreement with real range sizes (Table 3). All R^2^ of balance thresholds for models were higher than 0.919; LPT-derived models were similar with R^2^ higher than 0.894. Considering slope and intercept values, ES models met all criteria for use in absolute analysis of range-size relations: i) high R^2^; ii) intercept close to zero and iii) slope ≈1 (Table 3). All high performance models presented slopes higher than 1.0, suggesting over-estimation of absolute range-size (Table 3, Fig 8). Nevertheless, MXDEF with all variables had a close to 1.0 slope, and higher R^2^ among the high-performance models (Table 3, Fig 8). Considering all possibilities MXLQ with 4 PCA axes thus presented the best combination of all criteria with best absolute prediction of range-size (Table 3, Fig 8).

**Table 3:** Relationship between real and predicted range sizes based on a linear regression model. *a* is the intercept, *b* is the slope of the regression. R^2^ is the coefficient of determination. R^2^ values higher than 0.8 are highlighted. Higher R^2^ associated with *a*≈0 and *b*≈1 denotes best models for range-size prediction.

		LPT			Balance		
Environm. Variables	Modelling procedures	*a*	*b*	R^2^	*A*	*b*	R^2^
All	ES	107.4087	0.7586	0.974	107.4087	0.7586	0.974
All	Mahal	-103.1712	0.9338	0.954	129.8441	0.8254	0.943
All	MXLQ	261.7759	1.5257	0.955	584.5047	1.1697	0.958
All	MXDEF	169.613	1.3059	0.961	590.3274	1.0117	0.962
All	SVM	145.9529	1.5522	0.939	716.1815	1.1428	0.949
6 PCA	ES	234.4172	1.0185	0.972	234.4172	1.0185	0.972
6 PCA	Mahal	956.7514	1.4758	0.943	1071.594	1.1754	0.942
6 PCA	MXLQ	484.8208	1.4689	0.940	773.1833	1.16	0.959
6 PCA	MXDEF	416.2603	1.4710	0.945	752.6322	1.1566	0.958
6 PCA	SVM	676.0404	1.7828	0.901	1261.578	1.1252	0.919
4 PCA	ES	291.2412	1.1855	0.971	291.2412	1.1855	0.971
4 PCA	Mahal	628.8269	1.7099	0.926	1142.131	1.2586	0.954
4 PCA	MXLQ	682.4333	1.5067	0.943	927.4211	1.2327	0.954
4 PCA	MXDEF	658.1231	1.5100	0.940	922.4394	1.237	0.953
4 PCA	SVM	1040.6258	1.7033	0.894	1306.712	1.2166	0.936

## Discussion

Collinearity decreases the efficiency and increases the uncertainty of species distribution models. Nevertheless, the intensity of this effect among the algorithms ran contrary to our original hypothesis: More complex models behaved better than simple envelope models, which turn out to be highly sensitive to collinearity effects. No comprehensive test of these effects exists in the literature except Dormann et al. [35], who “…hesitantly conclude from our analysis that collinearity is a lesser problem than overfitting … or data uncertainty”. Based on a larger set of possible algorithms under evaluation, we conclude differently: collinearity exerts a strong effect, but these effects are higher on the simpler algorithms. Moreover, data uncertainty clearly remains as an important issue, but its interaction with collinearity may also be highly relevant. A positive message at this point lies in the relative efficiency that those complex models both described the spatial distribution of the virtual species and estimated its correct range-size. This supports its use both for theoretical discussions as for practical or applied questions.

Complex models are prone to overfitting as much as we define complex models as those with a larger number of parameters. Increased local fitting in some parts of the species’ range may decrease accuracy of prediction on other parts of the range [16,98]. Nevertheless, and despite these problems, complex models functioned better in face of collinearity. It is not easy to explain these differences, but complex methods clearly produced best fit to the data. The fitting process appears to be efficient in dealing with increased instability, and consequent uncertainty, related to the correlational structure of the independent variables. Difference in performance between collinear and not-collinear environmental datasets were low for methods such as Maxent, which may support the claim that the fitting process takes advantage of existing collinearity in finding a best set of parameters. Moreover, our results show that most of the problems with low performance algorithms are related to higher omission rates.

Overfitting is known to be a possible problem in Maxent models [28], and the algorithm had explicit ways to deal with this problem [99,100], but this effect may decrease model performance [77]. Use of MXLQ or other forms to constrain numbers of parameters [57], offers ways to control overfitting. Our results do not support the existence of large overfitting effects on model performance, but we did not explore spatial and environmental bias in sampling, or transferability problems that may emerge. Further evaluations may include other sampling schemes designed to mimic more accurately such effects.

Algorithm choice is considered as the most important source variation among SDM’s in recent studies [8,101]. Our results support this view, but we found that SVM and Maxent presented higher performance. Many other studies have also shown that Maxent models show lower uncertainty and may be considered the most reliable option [81,82,95,96,102]. A large number of current applications of SDM comprise very large sets of species to be evaluated, and observed idiosyncrasy among species may represent a problem. In such cases, it is difficult to perform a close look to individual responses and, in a worst scenario, very distinct choices applied to different species may lower the confidence on the generality of some results or on the comparisons we made [103]. Otherwise, the deeper understanding of Maxent omission/commission errors presented here and in other publications [100,104] may also support the best use of this tool.

Variable choice is widely considered as key for successful SDM application [105]. Many studies try carefully to describe these choices based on known ecology of focal species [106–108]; others simply used all information available [86,102,109]. This dichotomy is explained partly by constraints related to study objectives: studies dealing with a few species could strength their results by careful choices based on available ecological information (if it exists). Otherwise, no one expects that a single choice will cover the best possible option in studies dealing with large sets of species [8,58,110,111]. It is almost impossible to expect that individual setting of variables and parameters could be done in those large dataset studies [8,42]. Moreover, Araujo & Peterson [112] showed that both omission and commission errors may be affect by model misspecification, which is often caused by the absence of an important environmental factor during model fit. Use of all environmental variables has been criticized based on collinearity effects on model building. We showed that this is not a problem for modelling techniques such as Maxent and SVM. Otherwise, we consider that the best practice is to include all possibly relevant variables in the PCA and use a set of PCA-derived variables to provide surrogates for the ecological process that constrain species’ distribution. Nevertheless, our results also show that the use of PCA-derived variables may increase Overprediction rates, especially for narrow tolerance species. Obviously, these results are also dependent on the particular choice of threshold by the practitioner (please see Leroy et al. pre-print paper https://www.biorxiv.org/content/early/2018/05/11/235770) Even if, the composite TSS measure support the general view of the advantage of the use of PCA-derived variables, we consider that its use deserves special attention and study-purpose adjustments. Decoupling TSS in a more detailed picture showing the difference in over- and under- prediction rates will help to the user to avoid mis-interpretation and provide a much better description of the limits of its use.

We deliberately designed our virtual species to be heterogeneous and show substantial between-species variation of the ecological niche, to allow evaluating how different species characteristics affect prediction ability of algorithms. Our results support the view that the so called “idiosyncrasy” in species responses plays an important role in SDM performance: for example, we observed that some species always show lower prediction ability, regardless of algorithm choice. This was true even after controlling the prevalence effects on the evaluation metrics, which is especially important considering that prevalence was suggested as the hidden causes of those variations [113]. Species with restricted distributions presented higher TSS values, but in some cases prediction ability of different algorithms varied substantially. Idiosyncratic responses are especially important considering that many previous papers evaluating algorithm performance used small number of species [35] or species with similar environmental requirements from just one taxonomic group [114,115], which are prone to such effects.

The above mentioned general patterns of “idiosyncratic” species responses could be predicted based on some individual species characteristics that are expected to affect SDM performance. Thus, we refuse to maintain this as true idiosyncrasy. Our simulation design allows to directly evaluate the most important issues named (i) prevalence (partially under the label of tolerance in the environmental space), (ii) position in the ecological and geographical space and (iii) sample-size. Additionally, we specially devise the (iv) shape of species responses to environmental variables as an important issue. From these, the position in ecological/geographic space may be the more “idiosyncratic” in nature, but we may provide further insights on how its effect could be predicted. We now go deeper in each of these points.

Sample prevalence was considered an important predictor for model performance [116]. Narrow distributed species are expected to provide better models [82,117], nevertheless this is possibly a by-product of the overlooking of the spatial dependency of sample records [118]. The smallest is the range of the species, the farther are the mean distance between observed presence and absences used to calculate common metrics derived from the confusion matrix, such as AUC and TSS [93,118]. Nevertheless, our approach is not subjected to this flaw because we evaluate the models against to “real” virtual species distributions. We found only a slightly better performance in models for specialist species. Two simple methodological explanations could be devised. First, large ranges had also large borders. As previously observed, our approach allows to source-sink process that are expected to occur at distribution borders. The other explanation is simply the lack of discrimination due to environmental drivers for a species that occur in larger areas. As showed by the variation observed in distribution of our controlled virtual species (shown in Fig 3), range-size is not an obvious surrogate of species tolerance of environmental factors. In fact, a large variation among species with equal tolerances is caused by differences in the availability of those preferred climatic-environments in the geographical space. Thus, range-size should be carefully analyzed in such cases.

Position in ecological/geographic space effects are also affected by this discrepancy between tolerance in ecological space and range-size in geographical space. For example, a species with its mean optimal conditions in the Pantanal wetlands in Brazil, will probably have very little differences between broad- and narrow-tolerance in ecological space. This occurs because the cells in this region exhibit a high environmental similarity among themselves. Thus, position in the ecological/geographic space may indirectly determine species prevalence with consequences as discussed earlier. This effect is best described as the pattern of spatial autocorrelation of the different regions (in this case biomes in Brazil) and was also suggested as an idiosyncrasy generator [24,119].

Species responses to environmental variables plays an important role in determine the efficiency of SDM algorithms [69]. Our virtual species were built under only one response type, named Gaussian response, which may be well suited for general ecological theory [120], but will not answer for broad array of possibilities in real world. Previous studies show that some response types [69] may be associated to poor predictions for GLM/GAM models. Maxent is now viewed as more similar to a special kind of GLM methods, named Poisson regression [121], but it has a high flexibility and probably can account for linear relations, as well as it did for the non-linear relations in our study. Nevertheless, the case for different species responses and its effects on model efficiency is still open; with the need of a better evaluation of realistic cases were the same virtual species (same centroid and tolerance in environmental space) respond with different forms to different environmental variables. From another point of view, our choice to set occupancy thresholds at 10 and 90 percent reflects stable contiguous niches, which produce similar stable contiguous distribution in the geographical space [122]. For example in mountainous areas some species are restricted to rare patches of suitable habitat and so have extremely low occupancy [*e*.*g*. bee distribution in 123]. Nevertheless, our results still apply for a large range of species, and the effect of the threshold may be small.

Finally, we show that even models with low accuracy to predict suitability of individual cells may provide meaningful information on the estimation of range-size, a key species-trait for macroecological studies. This result is important for, at least, two broad uses of SDM. First, is to evaluate biogeographic or macroecological hypotheses build to explain broad patterns in species richness, range-size/abundance relations, or evolutionary patterns [124,125]. Our results suggest that most the algorithms may produce reliable information as surrogates for species range-size. The other use is more controversial. Range-size is one of the more important metrics determining the result of the evaluation of vulnerability according to IUCN methodology [126] and are used world-wide as criteria to prioritization of conservation efforts. SDM was considered as a possible surrogate for extent of occurrence (EOO), which could inform species categorization under IUCN categories [85,127], but there are also claims to use SDM to estimate area of occurrence (AOO) [128,129]. Our results support that all methods may provide accurate estimations of the absolute range-size and help inform species evaluation under the IUCN framework.

## Supporting information

S1 Table Basic parameters used to model virtual species response to original climatic variables. Identifier, geographic position of the simulated centroid and the centroid in the environmental space defined by original bioclim variables (b1: annual mean temperature; b2: mean diurnal range; b3: isothermality; b4: temperature seasonality; b5: max temperature of warmest month; b6: min temperature of coldest month; b7: temperature annual range; b8: mean temperature of wettest quarter; b9: mean temperature of driest quarter; b10: mean temperature of warmest quarter; b11: mean temperature of coldest quarter; b12: annual precipitation; b13: precipitation of wettest month; b14: precipitation of driest month; b15: precipitation seasonality; b16: precipitation of wettest quarter; b17: precipitation of driest quarter; b18: precipitation of warmest quarter; b19: precipitation of coldest quarter).
(DOCX)

S2 Table Basic parameters used to model virtual species response to PCA-transformed climatic variables. Identifier, geographic position of the simulated centroid and the centroid in the environmental space defined by the PCA-transformed variables of all simulated virtual species.
(DOCX)
