# Supplementary material for: Evaluating collinearity effects on species distribution models: An approach based on virtual species simulation
Source: PLoS One. 2018 Sep 11;13(9):e0202403. doi: 10.1371/journal.pone.0202403 (PMC6133275; doi:10.1371/journal.pone.0202403)
Supplement: S1 Table — Identifier, geographic position of the simulated centroid and the centroid in the environmental space defined by original bioclim variables (b1: annual mean temperature; b2: mean diurnal range; b3: isothermality; b4: temperature seasonality; b5: max temperature of warmest month; b6: min temperature of coldest month; b7: temperature annual range; b8: mean temperature of wettest quarter; b9: mean temperature of driest quarter; b10: mean temperature of warmest quarter; b11: mean temperature of coldest quarter; b12: annual precipitation; b13: precipitation of wettest month; b14: precipitation of driest month; b15: precipitation seasonality; b16: precipitation of wettest quarter; b17: precipitation of driest quarter; b18: precipitation of warmest quarter; b19: precipitation of coldest quarter). (DOCX) [file pone.0202403.s001.docx]

**S1 Table: Basic parameters used to model virtual species response to original climatic variables**

Identifier, geographic position of the simulated centroid and the centroid in the environmental space defined by original bioclim variables (b1: annual mean temperature; b2: mean diurnal range; b3: isothermality; b4: temperature seasonality; b5: max temperature of warmest month; b6: min temperature of coldest month; b7: temperature annual range; b8: mean temperature of wettest quarter; b9: mean temperature of driest quarter; b10: mean temperature of warmest quarter; b11: mean temperature of coldest quarter; b12: annual precipitation; b13: precipitation of wettest month; b14: precipitation of driest month; b15: precipitation seasonality; b16: precipitation of wettest quarter; b17: precipitation of driest quarter; b18: precipitation of warmest quarter; b19: precipitation of coldest quarter).

| Species | Longitude | Latitude | b1 | b2 | b3 | b4 | b5 | b6 | b7 | b8 | b9 | b10 | b11 | b12 | b13 | b14 | b15 | b16 | b17 | b18 | b19 |
| --- | --- | --- | --- | --- | --- | --- | --- | --- | --- | --- | --- | --- | --- | --- | --- | --- | --- | --- | --- | --- | --- |
| 1 | -58,9902 | -5,329 | 267 | 94 | 74 | 362 | 332 | 205 | 127 | 264 | 266 | 272 | 263 | 2255 | 296 | 59 | 45 | 864 | 243 | 345 | 860 |
| 2 | -39,0037 | -7,369 | 237 | 104 | 71 | 1141 | 310 | 165 | 145 | 238 | 229 | 250 | 221 | 1025 | 241 | 7 | 93 | 621 | 33 | 142 | 99 |
| 3 | -61,7434 | -26,030 | 223 | 142 | 53 | 4206 | 352 | 85 | 267 | 263 | 168 | 274 | 167 | 673 | 111 | 7 | 68 | 307 | 35 | 292 | 49 |
| 4 | -54,8093 | -30,822 | 186 | 115 | 50 | 3905 | 310 | 82 | 228 | 210 | 228 | 234 | 138 | 1388 | 136 | 94 | 11 | 391 | 314 | 340 | 328 |
| 5 | -54,5034 | -11,652 | 248 | 146 | 69 | 776 | 350 | 139 | 211 | 248 | 240 | 256 | 237 | 2015 | 325 | 1 | 77 | 958 | 14 | 529 | 52 |
| 6 | -44,7141 | -17,872 | 219 | 130 | 69 | 1750 | 299 | 112 | 187 | 232 | 194 | 235 | 192 | 1251 | 283 | 2 | 89 | 714 | 15 | 527 | 36 |
| 7 | -58,9902 | -18,178 | 250 | 127 | 67 | 2193 | 337 | 150 | 187 | 271 | 220 | 271 | 218 | 1189 | 186 | 25 | 53 | 507 | 106 | 477 | 138 |
| 8 | -41,8589 | -14,201 | 225 | 114 | 69 | 1359 | 304 | 140 | 164 | 234 | 204 | 236 | 204 | 665 | 139 | 3 | 86 | 371 | 14 | 338 | 14 |
| 9 | -40,9412 | -18,280 | 231 | 111 | 67 | 1527 | 313 | 148 | 165 | 245 | 211 | 250 | 211 | 1110 | 185 | 27 | 63 | 539 | 91 | 375 | 98 |
| 10 | -69,3913 | -10,224 | 249 | 114 | 73 | 746 | 319 | 164 | 155 | 254 | 238 | 256 | 238 | 1758 | 250 | 19 | 61 | 739 | 75 | 608 | 124 |
| 11 | -68,3716 | -37,468 | 141 | 163 | 50 | 5827 | 313 | -8 | 321 | 180 | 81 | 216 | 67 | 166 | 21 | 10 | 23 | 50 | 30 | 45 | 32 |
| 12 | -53,2798 | -11,873 | 249 | 150 | 66 | 838 | 357 | 133 | 224 | 251 | 238 | 256 | 236 | 2339 | 401 | 8 | 77 | 1131 | 34 | 590 | 49 |
| 13 | -46,0398 | -7,590 | 262 | 118 | 72 | 585 | 345 | 182 | 163 | 258 | 263 | 271 | 256 | 1214 | 210 | 2 | 77 | 589 | 14 | 136 | 182 |
| 14 | -53,0758 | -24,212 | 207 | 123 | 60 | 2931 | 306 | 101 | 205 | 241 | 168 | 241 | 167 | 1541 | 188 | 73 | 26 | 483 | 316 | 483 | 355 |
| 15 | -59,806 | 0,058 | 268 | 94 | 84 | 454 | 326 | 215 | 111 | 263 | 274 | 274 | 263 | 2018 | 323 | 84 | 45 | 853 | 286 | 286 | 773 |
