# Supplementary material for: Evaluating collinearity effects on species distribution models: An approach based on virtual species simulation
Source: PLoS One. 2018 Sep 11;13(9):e0202403. doi: 10.1371/journal.pone.0202403 (PMC6133275; doi:10.1371/journal.pone.0202403)
Supplement: S2 Table — Identifier, geographic position of the simulated centroid and the centroid in the environmental space defined by the PCA-transformed variables of all simulated virtual species. (DOCX) [file pone.0202403.s002.docx]

**S2 Table: Basic parameters used to model virtual species response to PCA-transformed climatic variables**

Identifier, geographic position of the simulated centroid and the centroid in the environmental space defined by the PCA-transformed variables of all simulated virtual species.

| Species | Longitude | Latitude | PC 1 | PC 2 | PC 3 | PC 4 | PC 5 | PC 6 |
| --- | --- | --- | --- | --- | --- | --- | --- | --- |
| 1 | -58,990 | -5,329 | 3,569 | -0,179 | -0,092 | 0,757 | 0,705 | 0,149 |
| 2 | -39,004 | -7,369 | 0,722 | 1,545 | 1,625 | 0,591 | -0,037 | -0,244 |
| 3 | -61,743 | -26,030 | -1,643 | 2,065 | -1,031 | -0,155 | -0,368 | 0,010 |
| 4 | -54,809 | -30,822 | -0,570 | -1,336 | -2,063 | 0,982 | -0,274 | 0,281 |
| 5 | -54,503 | -11,652 | 1,440 | 1,657 | 0,503 | -1,838 | -0,274 | -0,561 |
| 6 | -44,714 | -17,872 | 0,174 | 0,966 | 1,143 | -1,353 | -0,666 | -0,593 |
| 7 | -58,990 | -18,178 | 0,748 | 1,267 | -0,415 | -0,037 | -1,000 | 0,066 |
| 8 | -41,859 | -14,201 | -0,307 | 1,414 | 1,340 | 0,391 | -1,021 | -0,006 |
| 9 | -40,941 | -18,280 | 0,522 | 0,814 | 0,464 | 0,375 | -0,875 | -0,158 |
| 10 | -69,391 | -10,224 | 1,797 | 0,770 | 0,559 | -0,530 | -1,165 | -0,507 |
| 11 | -68,372 | -37,468 | -4,856 | 0,418 | -1,311 | 0,494 | 0,227 | 0,603 |
| 12 | -53,280 | -11,873 | 1,813 | 1,449 | 0,215 | -2,527 | -0,072 | -0,910 |
| 13 | -46,040 | -7,590 | 1,274 | 2,279 | 1,012 | 0,750 | 0,052 | 0,158 |
| 14 | -53,076 | -24,212 | 0,151 | -1,070 | -1,418 | 0,024 | -0,680 | 0,373 |
| 15 | -59,806 | 0,058 | 3,838 | -0,342 | 0,087 | 0,929 | 0,542 | 0,757 |
